# Impact of fibroblast activation protein on osteosarcoma cell lines *in vitro*

**DOI:** 10.3892/ol.2014.1788

**Published:** 2014-01-10

**Authors:** LIXIANG DING, LIN YE, JIANLI XU, WEN G. JIANG

**Affiliations:** 1 Cardiff University-Capital Medical University Joint Centre for Biomedical Research, Cardiff CF14 4XN, UK; 2 Metastasis and Angiogenesis Research Group, Institute of Cancer and Genetics, Cardiff University School of Medicine, Cardiff CF14 4XN, UK; 3 Department of Orthopaedic Surgery, Beijing Shijitan Hospital, Capital Medical University, Beijing 100038, P.R. China

**Keywords:** fibroblast activation protein/seprase, osteosarcoma, proliferation, adhesion, motility

## Abstract

Fibroblast activation protein (FAP) or seprase, which belongs to the group type II integral serine proteases, is an integral membrane serine peptidase. Previous studies have demonstrated that FAP has an effect on tumor growth, proliferation and invasion. However, the cellular functional role that FAP plays in osteosarcoma (OS) remains unknown. The aim of the present study was to investigate the activities of FAP in OS cell lines. The gene expression of FAP was knocked down through a hammerhead ribozyme transgene, and the various functions between the knockdown cells and their control cells were tested using a series of functional assays *in vitro*. The results indicated that knockdown of FAP markedly reduced the ability of cellular growth, matrix adhesion, migration and invasion in MG-63 and HOS cell lines compared with the control cells (P<0.05). In conclusion, FAP influences OS cells and may play a role in OS tumor progression and metastasis.

## Introduction

Osteosarcoma (OS) is the most prevalent form of aggressive primary malignant bone tumor with a high tendency to metastasize to the lung and occurs mainly in children and adolescents (1). Current optimal treatment consists of systemic multi-agent chemotherapy and surgical resection. The prognosis of OS patients has been markedly improved with the current optimal treatment. However, cases with metastases or an unresectable tumor have a poor prognosis. Therefore, there is a strong demand for early diagnosis and an improved therapeutic approach, which requires a comprehensive understanding of the molecular and cellular mechanisms of the disease. Human fibroblast activation protein (FAP, or seprase) is a 170-kDa homodimeric glycoprotein consisting of two 97-kDa subunits. FAP is an integral transmembrane protein belonging to the prolyl peptidase family with gelatinase and collagenase activity (2,3). Human FAP gene is located on chromosome 2q23 and the 760-aa FAP protein shows 48% sequence identity with dipeptidyl peptidase 4 (DPP-IV). FAP is expressed by reactive stromal fibroblasts in >90% of common types of human epithelial cancer, in the granulation tissue of healing wounds and in bone and soft tissue sarcomas (4–6). FAP and DPP-IV are known to form a hetero-oligomer in a proteolytic complex, which is involved in the invasion of tumor cells in collagenous matrices (7). Increasing evidence has suggested that the expression of the membrane-bound FAP in various solid tumors is associated with tumor growth and invasion and poor prognosis (2,8–12). This makes FAP an attractive subject when seeking a tumor biomarker or a potential therapeutic target for the disease (13). To date, the function of FAP in OS cells and implication in the disease progression remain unknown.

The current study sought to investigate the expression of FAP in OS cell lines and examine the association of this molecule with OS cell function. A FAP-knockdown cell model using hammerhead ribozymes was used to study the function of FAP *in vitro*.

## Materials and methods

### Immunohistochemical staining of FAP

Immunohistochemistry staining method of avidin-biotin complex (ABC) was used to test the protein expression of FAP in tissue sections. Paraffin samples of OS bone tissues (n=13) were sectioned (6-μm thick) and dewaxed using a series of gradient alcohol washes. Endogenous peroxidase activity was blocked with 0.3% hydrogen peroxide for 15 min prior to washes. Sections were then boiled, in a microwave, in antigen retrieval solution (pH 6.0) to retrieve antigen. Following washing in Tris-buffered saline (TBS) three times, the horse serum (Vector Laboratories, Ltd., Peterborough, UK) was added and the sections were incubated at room temperature for 30 min. The primary antibody (mouse anti-human FAP; 1:100; Santa Cruz Biotechnology, Inc., Santa Cruz, CA, USA), secondary antibody (multilink swine anti-mouse immunoglobulin) and ABC (Vector Laboratories, Ltd.) were added successively with 30, 30 and 45 min of incubation, respectively, and three TBS washes were performed in between. Absence of the primary antibody was used as a negative control. Diaminobenzidine chromogen (Vector Laboratories, Ltd.) was added to the sections and incubated in the dark for 5 min. Sections were then counterstained in Gill’s hematoxylin and dehydrated in ascending grades of methanol prior to clearing in xylene and mounting under a cover slip. Monoclonal mouse FAPα (ss-13; sc-100528) and anti-GAPDH (sc-32233) were obtained from Santa Cruz Biotechnology, Inc. Peroxidase-conjugated anti-mouse was purchased from Sigma-Aldrich (Poole, UK).

### Cell culture

OS cell lines, HOS and MG-63, were purchased from the European Collection of Animal Cell Cultures (Salisbury, UK). The cells were routinely cultured in Dulbecco’s modified Eagle’s medium/Ham’s F12 with L-Glutamine medium (PAA Laboratories, Yeovil, UK), supplemented with the antibiotics, penicillin and streptomycin and 10% fetal calf serum (PAA Laboratories) and incubated at 37.0°C, 5% CO_2_ and 95% humidity.

### Generation of FAP knockdown in OS cell lines

Anti-human FAP hammerhead ribozymes were designed based on the structure of FAP mRNA, generated using the Zuker RNA mFold program (14). The ribozymes were synthesized and cloned into a pEF6/V5-His-TOPO plasmid vector (Invitrogen Life Technologies, Paisley, UK). Ribozyme transgenes and empty pEF6 control plasmids were transfected into HOS and MG-63 cells individually, according to a previously reported procedure (15,16). Following transfection and blasticidin (5 μg/ml) selection, cells were subsequently cultured in medium with blastidin (0.5 μg/ml) to maintain the transfectants. The ribozyme transgene plasmids containing cells were routinely tested to confirm the knockdown of FAP expression at cDNA or protein level. Cells transfected with anti-FAP ribozyme and empty plasmid vector were respectively labeled as HOS^FAPRIB^ and HOS^pEF6^ for HOS cells and MG-63^FAPRIB^ and MG-63^pEF6^ for the transfected MG-63 cells.

### RNA extraction and reverse transcription-polymerase chain reaction (RT-PCR)

RNA was extracted from cells using the TRI reagent (Sigma-Aldrich). RT was performed using the iScript™ cDNA synthesis kit (Bio-Rad, Hercules, CA, USA). The following PCR conditions were used: Denaturing at 94°C for 40 sec; annealing at 55°C for 40 sec; and extension at 72°C for 60 sec. PCR was conducted over 30 cycles with an initial 5 min denaturing step (94°C) and a final 10 min extension step (72°C). PCR products were separated on a 2% agarose gel stained with ethidium bromide. The primer sequences used are provided in Table I.

### SDS-PAGE and western blotting

Proteins of each control or transfected cells were obtained following lysis. Equal amounts of each sample were separated on a 10% acrylamide gel. Following transfer from the gel onto a nitrocellulose membrane (Santa Cruz Biotechnology, Inc.), proteins were probed using the respective primary antibodies (FAP and GAPDH) at a concentration of 1:300, and specific peroxidase-conjugated secondary antibodies at a concentration of 1:1,000. Protein bands were documented using a gel documentation system (UVITech Ltd., Cambridge, UK).

### In vitro growth assay

Briefly, 2,000 cells were seeded into each well using three 96-well plates labeled as Day 1, 3 and 5. Following incubation for 1, 3 and 5 days, cells were fixed in 4% (v/v) formaldehyde and stained with 0.5% (w/v) crystal violet. The crystal violet stain was then extracted using 10% (v/v) acetic acid and cell density was determined by measuring the absorbance at a wavelength of 540 nm using an ELx800 spectrophotometer (BioTek Instruments, Inc., Winooski, VT, USA).

### In vitro Matrigel invasion assay

In the *in vitro* Matrigel invasion assay (17), a 24-well plate with Transwell inserts containing 8.0-μm pores (Becton-Dickinson, Franklin Lakes, NJ, USA) was first coated with 50 μg/insert of Matrigel matrix basement membrane (BD Biosciences, Oxford, UK). In total, 15,000 cells were seeded into Transwell inserts, followed by 72 h of incubation. After three days of incubation, cells which had invaded through the artificial basement membrane to the outside of the Transwell insert were fixed, stained and counted.

### In vitro Matrigel adhesion assay

In the *in vitro* Matrigel adhesion assay (18), a 96-well plate was precoated with 5 μg Matrigel per well. Briefly, 45,000 cells were seeded into each well. Following 45 min of incubation, non-adherent cells were removed by vigorous washing using TBS. Adherent cells were then fixed, stained and counted.

### In vitro migration/wound-healing assay

In the *in vitro* migration/wound-healing assay (19), a total of 40,000 cells were seeded in a 24-well plate and, upon reaching confluence, the medium was changed and the monolayer was scraped with a fine gauge needle to create a wound. The plate was placed on a heated plate to maintain a constant temperature of 37°C. Images of the cells were captured following wounding and every 15 min during 1.5 h with a digital camera (GXCAM-5; GT Vision Ltd., Suffolk, UK) attached to a microscope (Leitz DMIRB; Leica Microsystems Ltd., Buckinghamshire, UK) at ×200 magnification.

### Electric cell-substrate impedance sensing (ECIS)-based cellular motility assay

The 9600 model of the ECIS instrument (Applied BioPhysics, Inc., Troy, NY, USA) was used for attachment (adhesion) using a 96W1E plate, as well as a motility assay (wounding assay) (20–22). ECIS measures the interaction between cells and the substrate to which the cells are attached via gold-film electrodes placed on the surface of culture dishes. Following a stabilization, the same number of HOS^wt^, HOS^pEF6^, HOS^FAPrib^, MG^wt^, MG^pEF6^ or MG^FAPrib^ (80,000 per well) in the same volume of medium (200 μl) were added to each well. During the initial 3 h when cells were attaching to the bottom of the wells, impedance and resistance of the cell layer were recorded for the attachment ability analysis. After 10 h, when confluence was reached, the monolayer was electrically wounded at 6 V for 30 sec. Impedance and resistance of the cell layer were immediately recorded for a period of ≤20 h for the motility ability analysis.

### Statistical analysis

Experimental procedures were repeated independently at least three times. Statistical analysis was performed using the Minitab statistical software package (version 14; Minitab, Ltd., Coventry, UK). The two-sample t-test was used for normally distributed data, and data are presented as the mean ± standard error of the mean. P<0.05 was considered to indicate a statistically significant difference.

## Results

### Expression of FAP in OS cell lines and tissues

**Figure 1:**
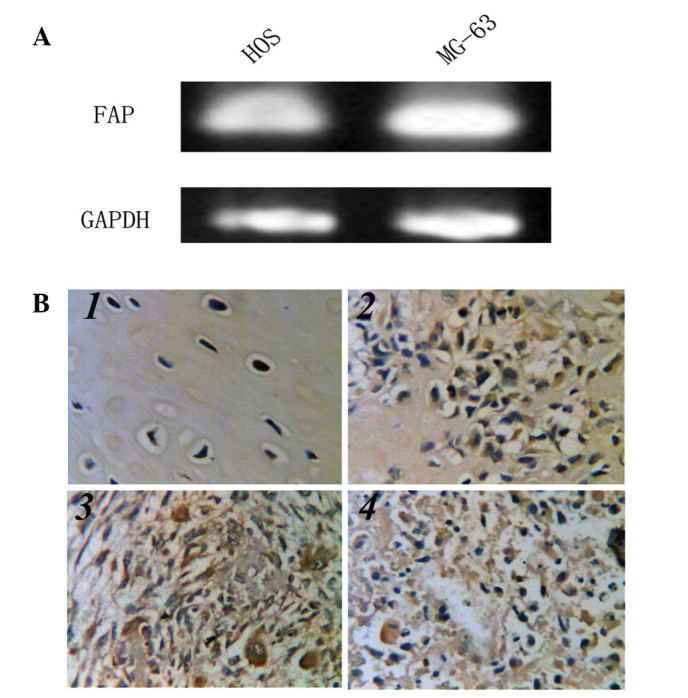
FAP expression in human OS cell lines and tissues. (A) Polymerase chain reaction analysis of FAP expression within a panel of OS cell lines. (B) Immunohistochemical staining of FAP in human OS tissues as follows: B1, background bone tissue; and B2, 3 and 4, OS tissues. All images were captured under a microscope (magnification, ×200). FAP, fibroblast activation protein; OS, osteosarcoma.

The presence of FAP was evident in the two human OS cell lines (HOS and MG-63) tested through RT-PCR (Fig. 1A). To investigate the biological function of FAP in OS, these cell lines were selected for knockdown studies.

To assess the expression pattern of FAP at the protein level, immunohistochemical staining of FAP was performed in the human OS tissues. Using a specific anti-FAP monoclonal antibody, FAP was detected in the cytoplasm of tumor cells, but was absent from osteocytes in the background bone tissues (Fig. 1B).

### Manipulation of FAP expression by ribozyme transgene

**Figure 2:**
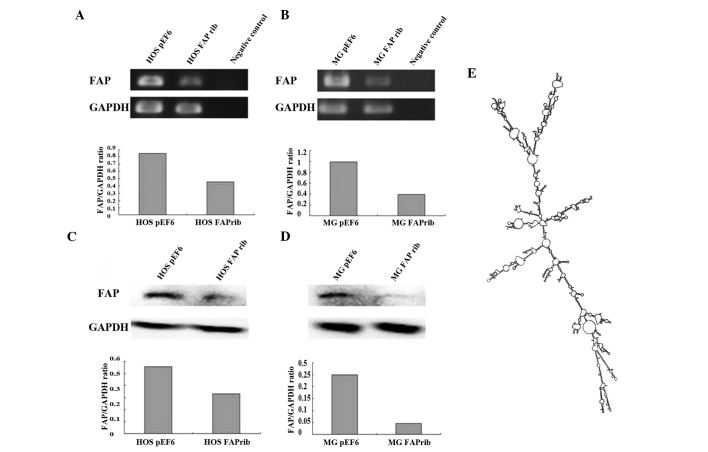
Knockdown of FAP in human osteosarcoma cell lines. Expression of FAP in HOS and MG-63 cell lines at the (A and B) cDNA level, as detected by polymerase chain reaction and (C and D) protein level, as detected by western blot analysis. The level of FAP was significantly lower in the knockdown cells compared with their pEF controls. (E) The secondary structure of FAP mRNA was used to design the anti-FAP ribozyme. FAP, fibroblast activation protein.

RT-PCR results demonstrated that FAP mRNA expression was successfully knocked down in HOS^FAPrib^ and MG^FAPrib^ cells in comparison with the level of expression in empty plasmid cells (HOS^pEF6^ and MG^pEF6^; Fig. 2A and B). Additionally, western blotting was used to probe for FAP protein levels in the control and transfected cell lines. Similar to the trends observed at the mRNA level, FAP protein was found to be highly expressed in all the control cell lines (HOS^WT^, MG^WT^, HOS^pEF6^ and MG^pEF6^) and expression of FAP protein exhibited a marked reduction in the transfected cell lines (HOS^FAPrib^ and MG^FAPrib^) (Fig. 2C and D).

### Knockdown of FAP reduces OS cell growth

**Figure 3:**
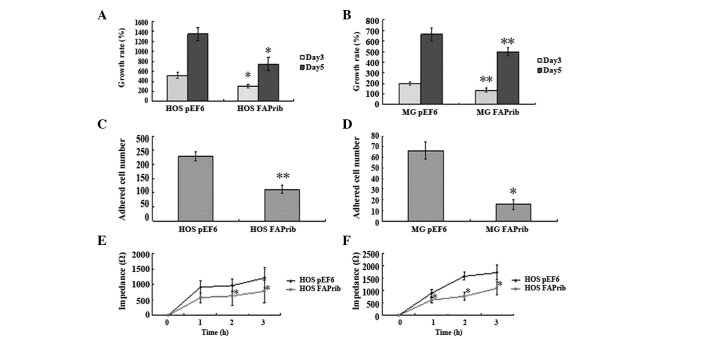
Cellular function tests of FAP in osteosarcoma cell lines. (A and B) Knockdown of FAP significantly reduced cell growth. (C and D) *In vitro* cell matrix adhesion assays were performed to assess the effect of FAP on the adhesiveness of osteosarcoma cells. (E and F) Knockdown of FAP significantly reduced cell adhesion, detected using an electric cell-substrate impedance sensing model system. FAP, fibroblast activation protein.

The *in vitro* tumor growth assay was used for the detection of change in growth caused by FAP knockdown in HOS and MG-63 cells. The same result was observed between these two cell lines when the growth rates were analyzed following a total 5-day incubation, which was that absence of FAP caused a low growth rate in OS cells, compared with their control cells. The results exhibited a statistically significant difference at day 3 (HOS^FAPrib^ vs. HOS^pEF6^: 137.7±19.8 vs. 199.1±15.6, P=0.027; and MG^FAPrib^ vs. MG^pEF6^: 301.9±31.2 vs. 516.7±54.8, P=0.007) and day 5 (HOS^FAPrib^ vs. HOS^pEF6^: 499.4±36.4 vs. 661.2±56.0, P=0.036; and MG^FAPrib^ vs. MG^pEF6^: 740.4±132.9 vs. 1345.7.2±132.3, P=0.009) (Fig. 3A and B).

### Loss of endogenous FAP results in low cell adhesion

To investigate the impact of the loss of FAP on the adhesive capability in OS cells, a 45-min period *in vitro* Matrigel adhesion assay and an ECIS assay were used. Cells adhering to the artificial Matrigel basement membrane were counted. Notably, the loss of FAP resulted in low adherence to the Matrigel in HOS and MG-63 cells (HOS^FAPrib^ vs. HOS^pEF6^: 111.0±15.0 vs. 227.3±15.6, P=0.009; and MG^FAPrib^ vs. MG^pEF6^: 15.5±4.4 vs. 66.2±8.1, P=0.025; Fig. 3C and D). In the attachment assay detected by ECIS, the same tendency was observed where knockdown cells showed a low adherence compared with their control cells following seeding for 1–3 h (HOS^FAPrib^ vs. HOS^pEF6^, P<0.05 at 2 and 3 h after seeding; and MG^FAPrib^ vs. MG^pEF6^, P<0.05 at 1, 2 and 3 h after seeding; Fig. 3E and F).

### Knockdown of FAP decreases cell invasion

**Figure 4:**
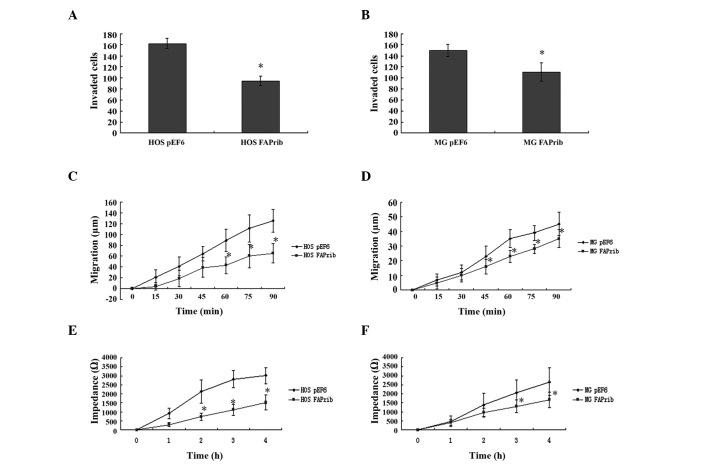
Knockdown of FAP decreases cell invasion, migration and motility in osteosarcoma cell lines using an electric cell-substrate impedance sensing model system. Knockdown of FAP significantly decreased cell (A and B) invasion, (C and D) migration *in vitro* and (E and F) motility. FAP, fibroblast activation protein.

The FAP-knockdown cells exhibited a relatively lower invasive capability than their control cells in the HOS and MG-63 cell lines (HOS^FAPrib^ vs. HOS^pEF6^: 95.0±8.5 vs. 162.3±8.8, P=0.013; and MG^FAPrib^ vs. MG^pEF6^: 110.7±16.2 vs. 150.2±11.3, P=0.034; Fig. 4A and B).

### Knockdown of FAP influences cell motility

The wounding assay compared the migration capabilities of OS cells between FAP-knockdown and control cells. The migration of HOS and MG-63 cells was reduced when FAP was absent during a 90 min incubation after the wounding (HOS^FAPrib^ vs. HOS^pEF6^, P<0.05 after 60 min; and MG^FAPrib^ vs. MG^pEF6^, P<0.05 after 45 min; Fig. 4C and D). The effect of FAP expression on cell motility was also assessed using an ECIS assay. Following wounding at 10 h, the record of impedance and resistance of the cell layer also showed the same result as the wounding assay, which was that FAP influenced cell motility (HOS^FAPrib^ vs. HOS^pEF6^, P<0.05 at 2, 3 and 4 h after wounding; and MG^FAPrib^ vs. MG^pEF6^, P<0.05 at 3 and 4 h after wounding; Fig. 4E and F).

## Discussion

FAP has been intensively investigated as a potential diagnostic or therapeutic target since it is overexpressed by activated stromal fibroblasts in malignant tumors and is absent in normal adult tissues and benign tumors (23–26). Since its identification, a number of previous studies have analyzed the localization and expression of this protease in diverse malignancies (27). FAP and DPP-IV expression is also found in bone sarcomas (5). However, the role of FAP in tumorigenesis and tumor growth, invasion and metastasis, as well as the exact molecular mechanisms, remain unknown. There is a clear discrepancy between FAP function in tumor promotion and suppression (12,28–30). Previously, Santos *et al* (26) showed that targeted gene disruption or pharmacological inhibition of FAP proteinase activity reduces the tumor growth in mouse models of lung and colon cancer. By contrast, other studies have suggested that FAP has tumor-suppressive activity (30,31). In the current study, the gene expression of FAP was knocked down through a hammerhead ribozyme transgene and the differences in the cellular functions between the knockdown cells and their controls were observed. The results of the current study indicated that knockdown of FAP markedly reduces the ability of cell growth, matrix adhesion, migration and invasion in MG-63 and HOS cell lines compared with the control cells.

The cancer-specific distribution of FAP makes it a novel therapeutic target in cancer treatment. While the function of FAP within malignancies remains poorly understood, efforts have been made to assess FAP as a therapeutic target, inhibiting its proteinase activity. FAP is transiently expressed in specific fetal mesenchymal tissues and is also expressed in certain disorders associated with activated stroma, including wound healing, rheumatoid arthritis, osteoarthritis, cirrhosis and pulmonary fibrosis (5,27,32). The effect of FAP on cellular functions and corresponding implications in bone development and remodeling remain poorly understood. The present study examined the function of FAP in OS cells and the implication in the disease progression. FAP is considered to promote tumor cell growth and proliferation (33). Chen *et al* (34) previously reported that FAP increases the invasion, proliferation and migration of ovarian cancer cells. The results of the present study revealed that bone sarcoma cell lines express FAP. The knockdown of FAP markedly decreases the *in vitro* growth, adhesion, migration and invasion of the OS cells.

FAP influences OS cells and may play a role in OS tumor progression and metastasis. Further investigation is likely to shed light on the relevant diagnostic and therapeutic potential of FAP in OS.

## Figures and Tables

**Table I:** Primers used for polymerase chain reaction.

Primer	Primer sequence, 5′-3′	Optimal annealing temperature, °C
FAP ribozyme		
2F	CTGCAGGTGGATCTCCTGGTCTTTGTTTCAATACTGATGAGTCCGTGAGGA	55
2R	ACTAGTAAATTAGCATATGTCTATCAAAACAATATTTCGTCCTCAGGACT	55
FAP		
F1	TCCCTTGCTAATTCAAGTGT	55
R1	AGAGCTTTAGCAATCTGTGC	55
F2	TGGAAAATGATTTGAAAAAT	55
R2	CTGTGTAGACAGACGCGTAA	55
GAPDH		
F8	GGCTGCTTTTAACTCTGGTA	55
R8	GACTGTGGTCATGAGTCCTT	55

FAP, fibroblast activation protein.
